# Analgesic and Anti-Inflammatory Effects of 80% Methanol Extract of* Leonotis ocymifolia *(Burm.f.) Iwarsson Leaves in Rodent Models

**DOI:** 10.1155/2018/1614793

**Published:** 2018-02-20

**Authors:** Asnakech Alemu, Wondmagegn Tamiru, Teshome Nedi, Workineh Shibeshi

**Affiliations:** ^1^Department of Pharmacology and Clinical Pharmacy, School of Pharmacy, College of Health Sciences, Addis Ababa University, Addis Ababa, Ethiopia; ^2^Food, Medicine and Healthcare Administration and Control Authority of Ethiopia (FMHACA), Addis Ababa, Ethiopia

## Abstract

**Background:**

Pain and inflammation are the major health problems commonly treated with traditional remedies mainly using medicinal plants.* Leonotis ocymifolia* is one of such medicinal plants used in folkloric medicine of Ethiopia. However, the plant has not been scientifically evaluated. The aim of this study was to evaluate analgesic and anti-inflammatory effects of the 80% methanol leaves extract of* Leonotis ocymifolia* using rodent models.

**Method:**

The central and peripheral analgesic effect of the extract at 100, 200, and 400 mg/kg dose levels was evaluated using hot plate and acetic acid induced writhing rodent models, whereas carrageenan induced paw edema and cotton pellet granuloma methods were used to screen anti-inflammatory effect of the extract at the same dose levels. Acute toxicity test was also done. Data were analyzed using one-way ANOVA followed by Tukey's post hoc test and *p* < 0.05 was considered significant.

**Results:**

The extract did not produce mortality up to 2000 mg/kg. All tested doses of the extract showed significant analgesic effect with maximum latency response of 62.8% and inhibition of acetic acid induced writhing. Maximum anti-inflammatory effect was recorded at 6 h after induction, with 75.88% reduction in carrageenan induced paw edema. Moreover, all tested doses of extract significantly inhibited the formation of inflammatory exudates and granuloma formation (*p* < 0.001).

**Conclusion:**

The study indicated that the extract was safe in mice and it has both analgesic and anti-inflammatory effect in rodent models.

## 1. Background

Pain is an unpleasant sensory and emotional experience associated with actual or potential tissue damage. Mostly, it is considered as one of the signs and symptoms of an illness and the most common reason for patients' medical visit [1]. Inflammation is also the most common adaptive response of the body. Both pain and inflammation involve a complex array of biochemical processes such as enzyme activation, inflammatory mediator release and extravasation of fluid, cell migration, and tissue damage and repair [2].

Despite the availability of sufficient drugs, the side effects of analgesic and anti-inflammatory agents, which include gastrointestinal upset, gastric ulcer, bleeding, and liver damage, are a major concern in clinical use. Because of this, the search for safe and effective newer agents is growing. As one of research areas, screening medicinal plants with claimed analgesic and anti-inflammatory effect may create the opportunity of discovering new compounds with more safety and efficacy [3]. Traditionally, different practices are routinely used to manage pain and inflammation in various countries. Herbal remedies are widely used in developing countries to manage pain and inflammation because of their cost, accessibility, and eco-friendly advantages [4].

In Ethiopia, a large number of plant specious are traditionally used to treat ailments associated with pain like headache, stomachache, and wound. Very few of these plants have been scientifically evaluated while most of them remain unexplored [3, 5]. The plant* Leonotis ocymifolia *(LO) belongs to the Lamiaceae family, also called Labiatae, and is characterized by a many-stemmed shrub, with average height of about 2.5 m [6, 7]. LO, locally known as Ras-kimir or Yeferes Zeng in Amharic [5], is indigenous to Eastern and Southern Africa [6].

Occasionally, it is used as an ascaricide and anticancer agent and as a remedy for ulcers and wounds. It is also reported that this plant is used in diabetes, hypertension, anemia, eczema, and other skin irritations [6]. It is also used for headache and ulcer of the neck [8], swelling [9], blackleg [10], hookworm, gout, and leishmaniasis. Moreover, it is reported to have antifertility [11] and antiemetic properties [12]. Moreover, the leaves of this plant are used to expel intestinal parasites from animals, particularly in the Bale Zone, Oromia region, Ethiopia [9, 13].

However, there were no studies so far investigating the use of LO for its analgesic and anti-inflammatory effects, although the plant is claimed to be used for this purpose by Ethiopian traditional healers [6, 8]. Therefore, the aim of this study was to investigate the acclaimed analgesic and anti-inflammatory effects of the leave extract of LO using various models in rodents.

## 2. Methods

### 2.1. Drugs and Chemicals

The drugs and chemicals used in the present study include carrageenan (Sigma Aldrich, Germany), aspirin and morphine (obtained from Ethiopian Pharmaceuticals Manufacturing), dexamethasone (Medico Labs, Lot E6A00, Syria), methanol (Carlo Erba, Italy), distilled water (Ethiopian Pharmaceuticals Manufacturing), Tween-80 and ammonia (Loba Chemie, India), glacial acetic acid, chloroform, and sulfuric acid (Fisher Scientific, UK), acetic anhydride (Park Scientific, UK), picric acid (Sigma Aldrich, Germany), and ferric chloride solution (Finkem laboratory reagent, India).

### 2.2. Materials and Instruments

Rotary evaporator (Heidolph, Germany), lyophilizer (OPERON, OPR-FDU-5012, Korea), digital plethysmometer (Ugo Basile, Cat number 7140, Italy), electronic balance (KERN-ALJ 220-4, Germany), hot plate, mini orbital shaker (SSM1-STUART), and tissue Drying Oven (Medite-Medizintechnik, Germany).

### 2.3. Plant Material Collection and Authentication

In January 2017, the fresh leaves of LO were collected from East-Showa Zone of Ada'a District, Denkaka Kebele, Oromia region, Ethiopia. Identification and authentication of the plant specimen were done at the National Herbarium, Department of Plant Biology and Biodiversity Management, Addis Ababa University. A voucher specimen was deposited with voucher number AS 001/2017 for future reference.

### 2.4. Experimental Animals

Experimental animals were obtained from the animal house of toxicology department of EFMHACA and the animal house of School of Pharmacy, Addis Ababa University. Healthy Swiss albino mice, either sex weighing 25–35 g and aged 6–8 weeks, were used for analgesic and acute anti-inflammatory tests. In chronic anti-inflammatory model, male albino Wistar rats weighing 180–220 g were used, whereas only female mice were used for the acute toxicity study.

Animals were kept in plastic cages at room temperature and on a 12 h light-dark cycle with access to food pellet and water ad libitum. They were acclimatized to the laboratory condition for a week before the experiments. The Organization of Economic Corporation and Development (OECD) guideline 425/2008 for the Care and Handling of Animals was followed in all of the experimental procedures [14, 15].

### 2.5. Preparation of Plant Extracts

The leaves were washed gently by rinsing with distilled water gently to remove debris and dust particles. Then, the leaves of LO were air dried under shade and pulverized using a mortar and pestle to a coarse powder which was used for the extraction. 250 grams of LO leaf was used and macerated in 600 ml of 80% methanol and the same volume of the solvent was used for successive extraction of the residues. To enhance the maceration process occasional shaking was carried out using mini orbital shaker with 120 rpm for 72 hrs at room temperature. Then, the extract was filtered first using muslin cloth and then using Whatman filter paper (number 1). Filtration and collection of the extract were done three times with the whole extraction taking 9 days. After the extraction, methanol was evaporated under vacuum using rotavapor at 40°C. The resulting solution was placed in a deep freezer operating at −20°C till it forms solid ice and then the remaining solvent (water) was removed using lyophilizer. After water removal, a light green black powder residue weighing 27.46 g was obtained, giving rise to a percentage yield of 11.44%. The powder residue was then stored at 4°C until use. The extract was dissolved in 2% Tween-80 in DW for the subsequent tests.

### 2.6. Acute Toxicity Study

Acute toxicity test was performed according to the OECD guideline 425/2008. Fasted female albino mice of 6–8 weeks were used for the toxicity study. First, a sighting study was performed to determine the starting dose, in which a single female mouse was given 2000 mg/kg of the extract as a single dose by oral gavage. Since no death was observed within 24 h, additional four mice were recruited for the extract treatment at the same dose. The animals were observed continuously in the first 4 h with 30 min interval and then for 14 consecutive days with an interval of 24 h for the general signs and symptoms of toxicities. These signs and symptoms include changes in skin, eyes, mucous membranes, somatomotor activity, behavioral pattern, salivation, diarrhea, weight loss, tremor, convulsions, lethargy, paralysis, food and water intake, and mortality. Based on the results of the acute toxicity test, three treatment doses of the extract were chosen: a middle dose, which was one-tenth of the maximum dose obtained during acute toxicity study; a low dose, which was half of the middle dose; and a high dose which was twice the middle dose.

### 2.7. Evaluation of Analgesic Activity of the Extract

#### 2.7.1. Acetic Acid Induced Writhing Method

The method of Arul et al. was used with slight modification [16]. Mice of either sex were divided into five groups with each consisting of six animals. Three groups were given different dose of the plant extract, while the control group was given a vehicle and the reference group was given 150 mg/kg of aspirin just one hour before 0.6% acetic acid (10 ml/kg, i.p.) administration [17]. Five minutes after the acetic acid injection i.p., the number of writhes was counted to determine analgesic activity of LO. The animals were placed in a glass jar individually and the contractions of abdominal muscles together with stretching of the hind limbs were cumulatively counted over a period of 30 minutes.

The percentage protection against writhing was taken as an index of analgesia [17, 18] and calculated using the following formula:

(1) %  Analgesic  Activity=Mean  writhing  count  control  group−Treated  groupMean  writhing  count  of  control  group×100.

#### 2.7.2. Hot Plate Method

This test consists of introducing a mouse into an open-ended cylindrical space with a floor consisting of a metallic plate which is heated at a constant temperature. This produces two behavioral components, which are measured in terms of their reaction times, namely, paw licking and jumping. These responses are considered to be supraspinally integrated [18]. Mice of either sex were divided into five groups, each consisting of six animals. All animals were fasted overnight. Three groups were given different doses of the plant extract (determined based on acute toxicity, p.o.), while one group was given a vehicle (control, p.o.) and the other group was given standard drug morphine (reference group, 20 mg/kg oral). The animals were placed on a hot plate maintained at a temperature of 55 ± 1°C [19]. Before the treatment, the reaction time of each animal was recorded. The latency to lick the paw or jump from the hot plate was noted as the reaction time. The reaction times were noted at 30, 60, 90, and 120 min. The cut-off time was considered as 15 s [20].

Percentage increase in reaction time or pain threshold inhibition was derived, using the formula [21]

(2) %  Elongation=Latency  test−Latency  (control)Letency  (test)×100.

### 2.8. Evaluation of Anti-Inflammatory Activity of the Extract

#### 2.8.1. Carrageenan Induced Mice Paw Edema

The acute anti-inflammatory activity of the extract was determined in mice according to the method of Winter et al. [22]. Mice were fasted for 12 h with free access to water until the experiment starts. Acute inflammation was produced by injection of carrageenan (1% w/v carrageenan in normal saline, 50*μ*l) in the right hind paw of the mice. Carrageenan was injected one hour after oral administration of the extract [23]. The inflammation was quantitated in terms of ml, that is, displacement of water by edema using a digital plethysmometer at times 0, 1, 2, 3, 4, 5, and 6 h after carrageenan injection [24]. The percent inhibition of edema was calculated in comparison to the control animals and was calculated using the following formula [25]:

(3) $$\begin{array}{c} \text{\% inhibition}=\frac{\text{Mean paw volume }\left(\text{control group}\right)-\text{Mean paw volume (treated group)}}{\text{Mean paw volume (control group)}}\times \text{100}. \end{array}$$

#### 2.8.2. Cotton Pellet Induced Granuloma Method

The method previously described by Meier et al. [26] was used with some modifications to assess the transudative and proliferative (granulomatous) components of chronic inflammation. Male albino Wistar rats (180–220 g) were fasted for 12 h with free access to water until commencement of the experiment. The control, reference, and test groups of rats received 2% Tween-80, dexamethasone (0.5 mg/kg p.o.) [27], and extracts, respectively.

Sterile cotton pellet weighing 10 ± 1 mg was prepared by rolling of a cotton piece of 10 mg and sterilized by autoclaving for 30 min at 120°C under 15 lbs pressure. Twenty minutes after treatment with the reference drug and extracts, the rats were anesthetized with diethyl ether and subcutaneous tunnel was made aseptically using blunted forceps in both sides of previously shaved groin region of each rat. Two sterilized cotton pellets weighing 10 ± 1 mg each were then implanted bilaterally in the subcutaneous tunnel and sutured with chromic catgut (0/4 metric-1/2 circle). Treatment with the reference drug (dexamethasone) and extracts continued for seven consecutive days (p.o., once a day). On the 8th day, the rats were sacrificed with diethyl ether anesthesia; thereafter, the pellets surrounded by granuloma tissue were dissected out carefully and freed from extraneous tissue. The wet weight of the cotton was taken immediately after removal and then dried at 60°C for 24 hrs and the net dry weight, that is, after subtracting the weight of the cotton pellets, was determined.

The exudate amount (mg), granulation tissue formation (mg), and percent inhibition of exudate and granuloma tissue formation were calculated according to the formula given below [28]:

(4) Exudates  inhibition  %=1−Exudates  in  treated  groupExudates  in  control  group×100,Granuloma  inhibition  %=1−Granuloma  in  Treated  groupExudates  in  control  group×100,

where

(5) Measure  of  Exudates=immediate  wet  weight  of  pellet−Constant  dry  weight  of  pellet,Measure  of  granuloma=Constant  dry  weight  of  cotton−Initial  weight  of  cotton  pellet.

### 2.9. Preliminary Phytochemical Screening

Phytochemical screening was carried out to confirm the presence or absence of secondary metabolites, which may be responsible for analgesic and anti-inflammatory effect, such as alkaloids, steroidal compounds, phenolic compounds, tannins, saponins, flavonoids, cardiac glycosides, and anthraquinones using standard procedures as stated in Trease and Evans as well as Abraham et al. [29, 30].

### 2.10. Statistical Analysis

The data were analyzed using SPSS version 20.0 for Windows. The experimental results are expressed as mean ± standard error of the mean (SEM) and statistical analysis was carried out using one-way analysis of variance (ANOVA) followed by Tukey's post hoc test for multiple comparisons among treatment groups. *p* value < 0.05 was considered statistically significant at 95% confidence interval. The analyzed data were then presented using tables. Linear regression was also used to determine dose-response relationships.

## 3. Results

### 3.1. Acute Toxicity Study

The acute oral toxicity test of 80% methanolic leaf extract (80 ME) of LO at the limit dose of 2000 mg/kg did not show gross behavioral changes, toxic effects, or mortality within the 24 h and the 14 days of observation. Therefore, the oral LD_50_ of crude 80 ME is greater than 2000 mg/kg in mice based on OECD 425/2008 guideline [15]. Hence, the result suggests that the extract is safe.

### 3.2. Analgesic Activity

#### 3.2.1. Acetic Acid Induced Writhing Assay

All test doses of the extract significantly (*p* < 0.001) reduced the acetic acid induced writhing in mice. Compared to the lower doses of the plant extract, the 400 mg/kg dose of LO (LO400) showed analgesic effect against acetic acid induced writhing response in mice (*p* < 0.001). Aspirin (150 mg/kg) also showed a potent analgesic effect (*p* < 0.001) compared to the 100 mg/kg and 200 mg/kg dose of the extracts. However, the LO400 has a comparable analgesic effect with that of aspirin.

**Figure 1:**
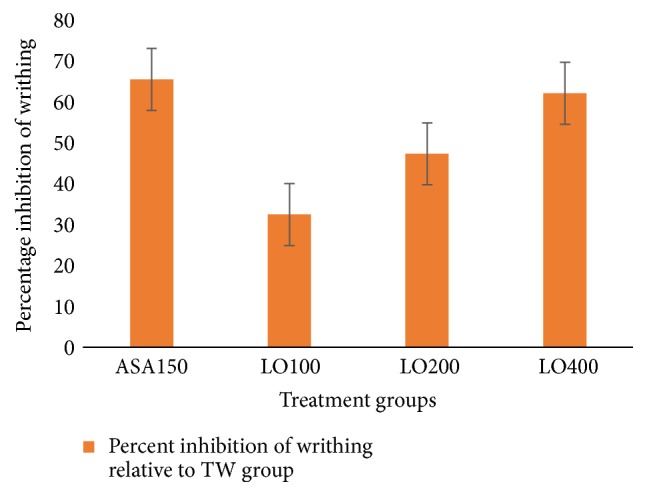
Percentage inhibition of 80% methanol leaf extract of* Leonotis ocymifolia* on acetic acid induced writhing model in mice. TW (10 ml/kg); ASA, aspirin (150 mg/kg); LO100, extract (100 mg/kg); LO200, extract (200 mg/kg); LO400, extract (400 mg/kg).

The 80% methanol extract of LO produced a significant (*p* < 0.001) analgesic effect against acetic acid induced writhing at the dose of 100, 200, and 400 mg/kg; the inhibitions were 32.8%, 47.9%, and 62.8% (Figure 1). Moreover, this effect of the extract was in dose dependent manner (*R*^2^ = 0.8628).

#### 3.2.2. Hot Plate Assay

**Figure 2:**
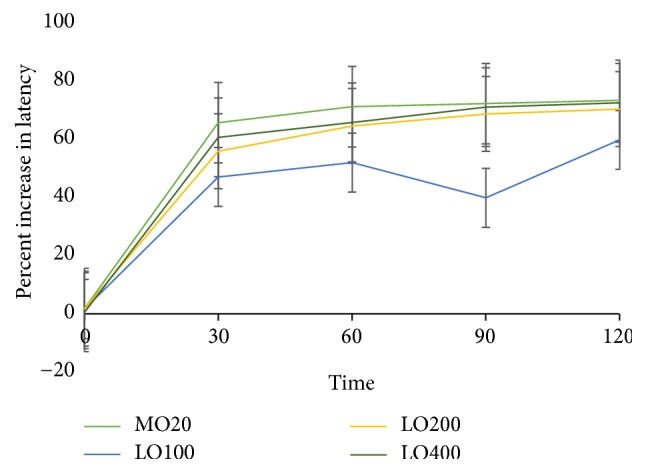
Percentage increase in latency time of 80% methanol extracts of* Leonotis ocymifolia* relative to negative control (TW) based on hot plate method latency time in mice. TW, Tween-80 (2%); MO, morphine (20 mg/kg); LO100, extract (100 mg/kg); LO200, extract (200 mg/kg); LO400, extract (400 mg/kg).

The LO extract significantly (*p* < 0.001) delayed the reaction time of mice to hot plate thermal stimulation at all dose levels with maximum effect at 120 min (Figure 2). LO400 and morphine produced a comparable maximum analgesic effect at 120 min (72.84%). The percent inhibition of 100, 200, and 400 mg/kg of LO was 59.3%, 69.9%, and 72.0%, respectively, at the 120 min point in dose dependent manner (*R*^2^ = 0.872). Intergroup comparison demonstrated that a significant delay in the response to the hot plate thermal stimulation was observed in the 200 mg/kg compared with 100 mg/kg (*p* < 0.001 from 30 min to 120 min), the 400 relative to 100 mg/kg (*p* < 0.001 from 30 min–120 min), whereas the 20 mg/kg morphine demonstrated potent analgesic effect compared with the 400 mg/kg (*p* < 0.001 from 30 min to 90 min and *p* < 0.05 at 120 min).

### 3.3. Anti-Inflammatory Activity

#### 3.3.1. Carrageenan Induced Paw Edema

**Figure 3:**
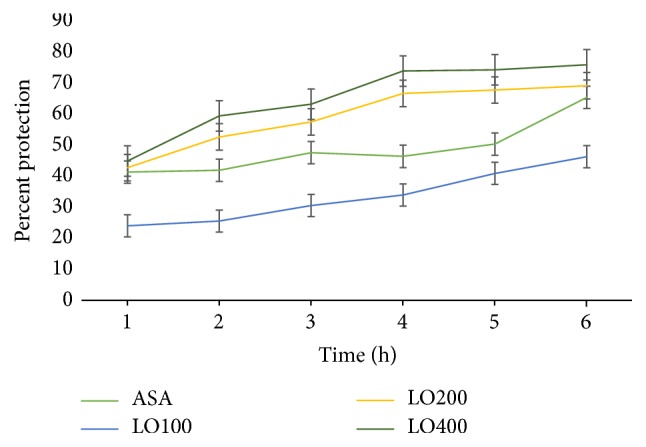
Percentage inhibition of 80% methanol extract of* Leonotis ocymifolia* on carrageenan induced paw edema model in mice. ASA, aspirin 100 mg/kg; LO100, extract (100 mg/kg); LO200, extract (200 mg/kg); LO400, extract (400 mg/kg).

All test doses of the ME80 showed a significant (*p* < 0.001) inhibition of paw edema from the 1st to the 6th h after induction (*p* < 0.001). The maximum anti-inflammatory effect of LO was observed at 6 h after induction at all doses, following a trend shown in Figure 3, with 46.3%, 69.13%, and 75.88% inhibition in dose dependent manner (*R*^2^ = 0.916). Intergroup comparison revealed that LO200 and LO400 got a comparable anti-inflammatory effect than the LO100, even higher than aspirin (150 mg/kg), throughout the observation period. However, aspirin has a comparable anti-inflammatory effect as that of LO100.

#### 3.3.2. Cotton Pellet Induced Granuloma

The ME80 of LO significantly (*p* < 0.001) prevented the formation of inflammatory exudates and granuloma mass at all tested doses. Intergroup comparisons revealed that the LO400 is relatively superior to the LO200 and LO100 in both exudate and granuloma inhibition (with *p* value ranging from <0.05 to <0.001). Furthermore, the anti-inflammatory effect of the LO was in dose dependent fashion (*R*^2^ = 0.558).

**Figure 4:**
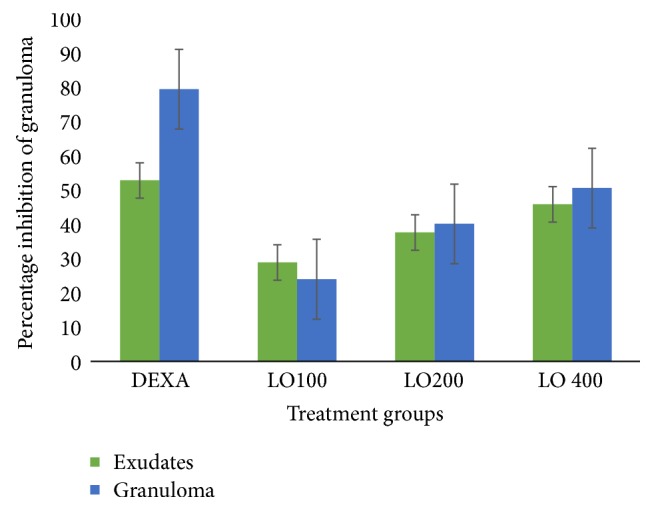
Percentage inhibition of 80% methanol extract of* Leonotis ocymifolia* on cotton pellet induced granuloma model in rats. Data is expressed as mean ± SEM, *n* = 6; TW, 2% Tween-80 (10 ml/kg); dexamethasone (0.5 mg/kg); LO100, extract (100 mg/kg); LO200, extract (200 mg/kg); LO400, extract (400 mg/kg).

On the other hand, 28.91%, 37.68%, and 45.91% inhibition of inflammatory exudate and 24.03%, 40.18%, and 50.65% reduction of granuloma were recorded at 100 mg/kg, 200 mg/kg, and 400 mg/kg doses, respectively, while, dexamethasone showed 52.89% and 79.54% inhibition of exudates and granuloma, respectively (Figure 4).

#### 3.3.3. Phytochemical Screening

Preliminary chemical screening of aqueous extract of the leaves of LO showed the presence of saponins, alkaloids, flavonoids, tannins, terpenoids, and phenols.

## 4. Discussion

*Leonotis ocymifolia* is a folkloric herbal medicine which has been used for the treatment of pain and inflammation in Ethiopia [9]. However, its pharmacological analgesic and anti-inflammatory effects have not been scientifically validated. Therefore, the aim of the present study was to investigate the analgesic and anti-inflammatory effects of the 80% methanol extract of LO rodent models.

The LO effectively inhibited acetic acid induced pain. Acetic acid induced writhing mice model is used to study the peripheral analgesic activity of test substances. The inflammatory pain in this model is induced by capillary permeability and reduced nociceptive threshold because of stimulation of nociceptive fibers of the nervous terminal [31]. It may also induce pain by increasing the release of PGE_2_ and PGF_2*α*_ at the peritoneal receptors [32, 33]. Thus, the analgesic effect of LO may be due to interference of these nociceptive targets. On the other hand, LO delayed the latency time for hot plate model. This model, which relies on nociceptive reaction against thermal stimuli, is well-established for detection of opiate (narcotic) analgesic property for centrally acting analgesics [34]. Therefore, the result may suggest that the extract may also have a central analgesic effect.

In carrageenan induced model, LO reversed carrageenan induced inflammation and swelling. This experimental model was employed to evaluate the effect of the extract on acute inflammation [32, 35]. Carrageenan is an agent of choice for testing anti-inflammatory drugs since it is devoid of antigenic reaction and systemic effects with high degree of reproducibility [36]. It involves a biphasic release of various types of chemical mediators of inflammation such as histamine, serotonin, bradykinin, and prostaglandins, which cause pain and fever [36–38]. Thus, the anti-inflammatory effect of LO may involve interferences on such processes, particularly at the later stage.

However, the cotton pellet induced granuloma model was used to test the effect of the extract in chronic inflammation [39]. This model is commonly used to assess the transudative and proliferative components of chronic inflammation [32, 40]. The weight of wet cotton pellets correlates with exudative material and the weight of dry pellets correlates with the amount of granulomatous tissue [41]. Based on the findings, LO effectively inhibited granuloma formation, which indicates its effect against chronic inflammation.

Plants which have analgesic and anti-inflammatory activity contain mainly alkaloids, flavonoids, saponin, tannins phenolic compound, glycosides, coumarins, and triterpenoids chemical constituents [42–45]. Tannins, flavonoids, and saponins are well known for their ability to inhibit pain perception and anti-inflammatory properties due to inhibition of enzymes involved in inflammation, especially arachidonic acid metabolic pathway, and synthesis of prostaglandins [46, 47]. Tannins could affect the inflammatory response via free radical scavenging properties and inhibition of iNOS in macrophages [48]. Saponins, on the other hand, inhibit pain and inflammation via NO inhibition [49, 50]. Therefore, the presence of saponins, alkaloids, flavonoids, tannins, terpenoids, and phenols may be responsible for the analgesic and anti-inflammatory effects of LO.

## 5. Conclusion

The present study indicated that the 80% methanol leaf extract of* Leonotis ocymifolia* is safe in mice. In addition, it has both analgesic and anti-inflammatory activities in rodent models. The presence of saponins, alkaloids, flavonoids, tannins, terpenoids, and phenolic compounds was also confirmed in the extract.

## Data Availability

The datasets used and/or analyzed during the current study are available from the corresponding author on reasonable request.
